# 分散固相萃取-超高效液相色谱-串联质谱法测定水产品中5种硝基咪唑类和6种苯二氮卓类药物

**DOI:** 10.3724/SP.J.1123.2022.01005

**Published:** 2022-07-08

**Authors:** Xiao YANG, Yiwen WAN, Huawei HUANG, Wenwen SUO, Wei XIAO, Xiaoling LI

**Affiliations:** 1.湖南省水产科学研究所, 湖南 长沙 410153; 1. Hunan Fisheries Science Institute, Changsha 410153, China; 2.农业农村部渔业产品质量监督检验测试中心(长沙), 湖南 长沙 410153; 2. Fishery Products Quality Supervision and Testing Center, Ministry of Agriculture and Rural Affairs, Changsha 410153, China

**Keywords:** 超高效液相色谱-串联质谱, 分散固相萃取, 硝基咪唑, 苯二氮卓, 水产品, ultra-high performance liquid chromatography-tandem mass spectrometry (UHPLC-MS/MS), dispersive solid-phase extraction (dSPE), nitromidazoles, benzodiazepines, aquatic products

## Abstract

建立了分散固相萃取-超高效液相色谱-串联质谱法同时测定水产品中5种硝基咪唑类和6种苯二氮卓类药物残留的方法。样品用1%(v/v)氨水乙腈提取,提取液经十八烷基键合硅胶(C18)和*N*-丙基乙二胺(PSA)吸附剂净化,在45 ℃下用氮气吹至近干,用1 mL甲醇-水(1:9, v/v)溶液复溶,过0.22 μm尼龙-66滤膜后用超高效液相色谱-串联质谱测定。目标化合物采用Kinetex F5色谱柱(100 mm×3.0 mm, 2.6 μm)分离,以0.1%(v/v)甲酸水溶液和甲醇作为流动相进行梯度洗脱,在电喷雾离子源(ESI)、正离子扫描和多反应监测(MRM)模式下进行测定,基质匹配外标法定量。结果表明,5种硝基咪唑类和6种苯二氮卓类药物在8.5 min内完成色谱分离分析,目标物在0.5~20 μg/L范围内线性关系良好,相关系数均大于0.995,检出限和定量限分别为0.2~0.5 μg/kg和0.5~1.0 μg/kg。以草鱼、对虾和大黄鱼为样品基质,在3个不同的添加水平下,5种硝基咪唑类和6种苯二氮卓类药物的平均回收率为73.2%~110.6%,相对标准偏差(RSD)小于15%。本研究建立的方法具有简单、快速、灵敏度高和成本低等优势,可用于水产品中5种硝基咪唑类和6种苯二氮卓类药物的快速检测。该方法的建立为我国水产品质量安全相关监管部门同时监控水产品中硝基咪唑类和苯二氮卓类药物残留提供了技术支持。

## Abstract (en)

Nitroimidazoles (NMZs) are a crucial group of antibacterial compounds from a historical perspective. In the past, they were used for treating and preventing parasitic infections in fish. Benzodiazepines (BZDs) are second-generation sedative-hypnotics. Some fish farmers or vendors use them illegally to keep aquatic products fresh during the transportation of aquatic animals. Aquatic products are one of the most common food sources of protein and can be contaminated by NMZs and BZDs, which could impact humans through the food chain. Until recently, there was limited information on the simultaneous determination of NMZs and BZDs. Thus, it is critical to accurately quantify NMZs and BZDs for risk assessment and risk monitoring of food safety.

For the simultaneous determination of five nitroimidazoles and six benzodiazepines in aquatic products, a new approach based on the dispersive solid-phase extraction (dSPE) coupled with ultra-high performance liquid chromatography-tandem mass spectrometry (UHPLC-MS/MS) was developed. First, the samples were extracted with acetonitrile containing 1% (v/v) ammonium hydroxide, and the extracts were purified using dSPE with C18 and primary secondary amine sorbents. Second, the extracts were collected and dried at 45 ℃ under nitrogen flow. Finally, the extracts were redissolved in 1 mL methanol-water (1:9, v/v) mixture, filtered through a nylon-66 microfiltration membrane, and analyzed using UHPLC-MS/MS. The separation of compounds was conducted on a Kinetex F5 column (100 mm×3.0 mm, 2.6 μm) using gradient elution with 1% (v/v) formic acid aqueous solution and methanol as the mobile phase. The analytes were detected using MS/MS with positive electrospray ionization (ESI^+^) source under the multiple reaction monitoring modes. The matrix-matched external standard approach was used for quantitative analysis. The compounds of five nitroimidazoles and six benzodiazepines could be examined within 8.5 min. Under the optimal conditions, the standard curves were linear in the range of 0.5-20 μg/L, with the correlation coefficients exceeding 0.995. The limits of detection and limits of quantification were 0.2-0.5 μg/kg and 0.5-1.0 μg/kg, respectively. The average recoveries at three spiked levels in blank samples (grass carp, large yellow croaker, and prawn) ranged from 73.2% to 110.6%, with relative standard deviations of less than 15%. The developed approach is simple, sensitive, fast, and inexpensive. It can be used for determining five nitroimidazoles and six benzodiazepines in aquatic products.

硝基咪唑类药物(nitromidazoles, NMZs)是一种具有5-硝基取代咪唑杂环结构的化合物,常见的包括甲硝唑(metronidazole, MNZ)、地美硝唑(dimetridazole, DMZ)、洛硝哒唑(ronidazole, RNZ)、替硝唑(tinidazole, TNZ)等。NMZs对原生动物和厌氧菌引起的某些疾病具有治疗作用,在水产养殖中被用于预防和治疗寄生虫感染^[1,2]^。NMZs被过量施用于水产动物后,原药可能在水产品中残留。同时,原药在生物体内代谢迅速,其代谢产物为羟基化产物,主要包括羟基甲硝唑(MNZOH, MNZ的代谢物)和羟基二甲硝咪唑(HMMNI, DMZ和RNZ共同的代谢物)。NMZs具有潜在的致癌性、诱变性和遗传毒性,其代谢物由于保留了基本的咪唑环也具有类似的毒性^[1,3,4]^。该类药物滥用后易产生细菌耐药性,其在动物源食品中的残留对食品安全构成威胁。苯二氮卓类药物(benzodiazepines, BZDs)大多是1,4-苯并二氮卓的衍生物,其基本结构含有两个苯环和一个七原子杂环。BZDs属于第二代镇静安眠药,临床上常用的有20多种,具有镇静催眠、抗焦虑、抗惊厥、肌肉松弛和安定等多种药理作用。近年来,一些养殖户或商贩为了保持水产品的鲜活状态,在水产动物捕捞和运输过程中非法使用地西泮等苯二氮卓类药物,造成其在水产品中残留,严重危害人体健康。为控制NMZs和BZDs在动物源性食品中的残留,我国、欧盟和美国对以上两类药物的使用均进行了限定。我国农业农村部公告第250号规定洛硝达唑和替硝唑在食品动物中禁止使用。同时,我国《食品安全国家标准 食品中兽药最大残留限量》(GB 31650-2019)规定地美硝唑、甲硝唑和地西泮允许作治疗用,但不得在动物性食品中检出。欧盟将甲硝唑、地美硝唑和洛硝哒唑列入食品动物禁用化合物清单,对苯二氮卓类镇静剂推出了限量标准^[5,6]^。美国禁止在食品动物中使用硝基咪唑类药物^[7]^。因此,为了进一步加强水产品质量安全问题的监管,开发简单、灵敏、快速测定水产品中硝基咪唑类和苯二氮卓类药物的检测方法显得十分必要。

目前,检测NMZs或BZDs的方法主要有酶联免疫法(ELISA)^[8,9]^、气相色谱-质谱联用法(GC-MS)^[10,11]^、高效液相色谱法(HPLC)^[12,13]^、液相色谱-串联质谱法(LC-MS/MS)^[14⇓⇓⇓⇓-19]^。酶联免疫法适用于批量样品快速筛查,检测结果需要用其他方法确证;GC-MS一般需要衍生化,操作繁琐、耗时;HPLC灵敏度较低,对多组分化合物同时测定时分析时间长且易受杂质干扰。LC-MS/MS灵敏度高,选择性好,抗干扰能力强,适用于对基质复杂样品中多组分兽药残留的定性、定量检测。水产品种类繁多,基质较为复杂,含有较多的蛋白质、脂肪和色素等成分。检测水产品中NMZs和BZDs残留的关键之一是样品前处理过程必须充分地减少基质对待测目标物的干扰。当前国内外尚未发现同时检测NMZs和BZDs的文献报道,已有的研究报道仅见针对单一药物的检测,样品前处理主要采用固相萃取技术(solid-phase extraction, SPE)^[13,17,18,20]^或分散固相萃取技术(dispersive solid-phase extraction, dSPE)^[21,22]^进行富集和净化。SPE是一种应用于样品前处理的经典萃取技术,但也存在萃取装置、耗材成本高,萃取过程繁琐耗时,萃取吸附剂填料选择范围小等缺点^[23]^。dSPE是一种简单、高效、快速的前处理技术,近年来在动物源食品中NMZs或BZDs多残留检测中得到了较为广泛的关注和应用^[21,22,24,25]^。

本研究采用分散固相萃取技术,结合超高效液相色谱-串联质谱(UHPLC-MS/MS),建立了一种同时测定水产品中5种硝基咪唑类和6种苯二氮卓类药物残留的方法。该方法操作简单高效,灵敏度高,回收率好,适用于大批量样品的快速检测,可为水产品质量安全相关监管部门同时监控水产品中硝基咪唑类和苯二氮卓类药物残留提供技术支持。

## 1 实验部分

### 1.1 仪器与试剂

Triple Quad 5500+超高效液相色谱-串联质谱仪(美国SCIEX公司);超声清洗器(昆山市超声仪器有限公司);高速冷冻离心机(美国Thermo Fisher Scientific公司);涡旋混匀器(美国Scilogex公司);氮吹仪(美国Organomation公司);中沃超纯水仪(中沃水务环保科技有限公司); Kinetex F5色谱柱(100 mm×3.0 mm, 2.6 μm;美国Phenomenex公司)。

甲硝唑(纯度≥99.8%, CAS号:443-48-1)、地美硝唑(纯度≥99.5%, CAS号:551-92-8)、洛硝达唑(纯度≥99.1%, CAS号:7681-76-7)、羟基甲硝唑(纯度≥99.5%, CAS号:4812-40-2)、羟基二甲硝咪唑(纯度≥99.5%, CAS号:936-05-0)均购自德国Dr. Ehrenstorfer公司;地西泮(1 mg/mL, CAS号:439-14-5)、硝西泮(1 mg/mL, CAS号:146-22-5)、氯硝西泮(1 mg/mL, CAS号:1622-61-3)、奥沙西泮(1 mg/mL, CAS号:604-75-1)、氟西泮(1 mg/mL, CAS号:17617-23-1)、氯氮卓(1 mg/mL, CAS号:58-25-3)均购自美国Sigma-Aldrich公司;甲醇、乙腈(色谱纯,德国Merck公司);甲酸(LC-MS级,美国Sigma公司);氯化钠、无水硫酸钠(分析纯,上海国药集团股份有限公司);十八烷基键合硅胶吸附剂(C18, 40~63 μm)、*N*-丙基乙二胺(PSA, 40~63 μm)吸附剂(上海安谱实验科技有限公司);实验用水为超纯水(18.25 MΩ·cm)。

### 1.2 样品处理

#### 1.2.1 样品采集与制备

鲫鱼、鳊鱼、鲤鱼、草鱼、鲢鱼、乌鳢、对虾、大黄鱼等水产品购自长沙市水渡河农产品批发市场。按照《水产品抽样规范》(GB/T 30891-2014)附录B的要求进行样品的制备,制备好的样品于-18 ℃冷冻保存,备用。

#### 1.2.2 提取

称取2.0 g (±0.02 g)试样于50 mL离心管中,加入10 mL 1%(v/v)氨水乙腈、1 g氯化钠、3 g无水硫酸钠,涡旋混匀1 min,超声提取10 min, 8000 r/min离心5 min。收集上清液于另一50 mL离心管中。残渣加入10 mL 1%(v/v)氨水乙腈重复提取一次,合并两次上清液,待净化。

#### 1.2.3 净化

准确移取10 mL提取液至装有QuEChERS吸附剂(50 mg PSA、30 mg C18)的15 mL离心管中,涡旋混匀1 min, 10000 r/min离心8 min。吸取全部上清液于15 mL离心管中,45 ℃氮吹至近干。准确加入1.00 mL 10%(v/v)甲醇水溶液,涡旋混匀1 min溶解残渣,过0.22 μm尼龙-66微孔滤膜,供UHPLC-MS/MS测定。

### 1.3 标准溶液的配制

分别准确称取5种硝基咪唑类标准物质,用甲醇溶解,配制成100 mg/L的标准储备液。分别移取6种苯二氮卓类标准物质1.00 mL,置于10 mL容量瓶中,用甲醇稀释定容至刻度,配制成100 mg/L的标准储备液。分别移取适量上述标准储备液,用甲醇稀释配制成1.0 mg/L的混合标准中间液。

称取不含目标物的基质样品于离心管中,按照1.2.2和1.2.3节步骤进行提取和净化,得到空白基质溶液。移取适量混合标准中间液,用空白基质溶液配制成质量浓度为0.5、1.0、2.0、5.0、10.0和20.0 μg/L的系列混合标准工作液。

### 1.4 色谱-质谱条件

#### 1.4.1 色谱条件

Kinetex F5色谱柱(100 mm×3.0 mm, 2.6 μm);柱温40 ℃;流速0.35 mL/min;进样量5 μL;流动相A为0.1%(v/v)甲酸水溶液,B为甲醇。梯度洗脱程序:0~0.5 min, 10%B; 0.5~4 min, 10%B~95%B; 4~6 min, 95%B; 6~6.1 min,95%B~10%B; 6.1~8.5 min, 10%B。

#### 1.4.2 质谱条件

电喷雾离子源(ESI),多反应监测(MRM);喷雾电压:5500 V;离子源温度550 ℃;气帘气(curtain gas)压力241 kPa,碰撞气(collision gas)压力62 kPa,喷雾气压力(Gas 1)345 kPa,辅助加热气(Gas 2)压力379 kPa。优化后的其他质谱采集参数见表1。

**表 1:** 5种硝基咪唑类和6种苯二氮卓类药物的质谱分析参数

Toxin	ESI mode	Parent ion (m/z)	Product ion (m/z)	Declustering potential/V	Collision energy/eV
Metronidazole	ESI^+^	172.1	128.0^*^	75	21
			81.9		34
Dimetridazole	ESI^+^	142.1	96.1^*^	70	23
			81.1		35
Ronidazole	ESI^+^	201.1	140.1^*^	60	17
			55.1		32
Hydroxy metronidazole (MNZOH)	ESI^+^	188.1	126.1^*^	82	25
			123.0		19
Hydroxy dimetridazole (HMMNI)	ESI^+^	158.1	140.1^*^	70	18
			55.0		26
Diazepam	ESI^+^	285.2	193.1^*^	102	44
			154.1		40
Nitrazepam	ESI^+^	282.2	236.2^*^	100	36
			180.2		50
Flurazepam	ESI^+^	388.1	315.1^*^	110	35
			288.1		37
Clonazepam	ESI^+^	316.1	270.1^*^	112	36
			214.0		50
Oxazepam	ESI^+^	287.0	241.1^*^	100	32
			104.0		45
Chlordiazepoxide	ESI^+^	300.1	283.1^*^	95	22
			227.1		35

* Quantitative ion.

## 2 结果与讨论

### 2.1 分析条件的优化

#### 2.1.1 色谱条件的优化

硝基咪唑类药物和苯二氮卓类药物是一类中等极性的化合物,在C18色谱柱上具有较强的保留。Kinetex F5柱(100 mm×3.0 mm, 2.6 μm)是一款采用核-壳技术的五氟苯基丙基固定相色谱柱,非常适用于卤代、共轭、异构体或极性化合物的分离分析。对于液相色谱-串联质谱法,流动相的类型和性质是影响目标化合物分离度和响应的重要因素。本实验研究发现硝基咪唑类药物和苯二氮卓类药物在甲醇-水体系中比在乙腈-水体系中的响应值更高。采用甲醇-水体系作为流动相,在水相中加入2 mmoL/L的乙酸铵后,部分目标物的色谱峰出现峰展宽的现象,响应强度减小;而在水相中加入0.1%(v/v)甲酸后,目标物峰形尖锐对称,离子化效率更高,响应强度增大。考虑到目标分析物种类较多,化学性质差异较大,采用梯度洗脱程序,可有效缩短分析时间,改善峰形,减少拖尾,改进多组分目标物的分离效果。

因此,本实验最终确定选择0.1%(v/v)甲酸水-甲醇体系作为流动相,采用梯度洗脱程序进行分析检测,色谱条件见1.4.1节。5种硝基咪唑类和6种苯二氮卓类药物混合标准溶液(5 μg/L,以10%(v/v)甲醇水溶液配制)的MRM色谱图见图1。

**图 1:**
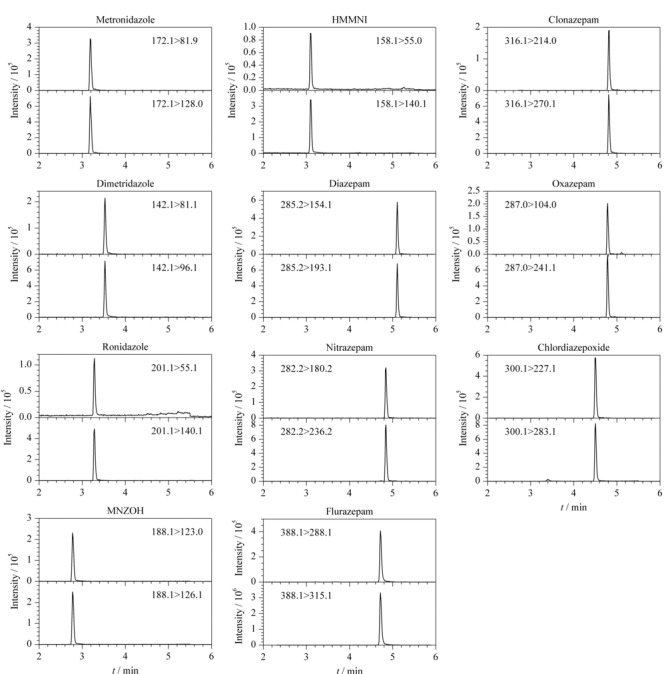
5种硝基咪唑类和6种苯二氮卓类药物混合标准溶液的多反应监测色谱图

#### 2.1.2 质谱条件的优化

将50 μg/L的硝基咪唑类和苯二氮卓类标准溶液通过针泵恒流进样方式注入质谱仪中,在正离子模式(ESI^+^)下进行一级质谱扫描(Q1 scan),获得目标化合物的母离子[M+H]^+^。然后使用子离子扫描(product ion scan)对获得的母离子进行二级质谱扫描,确定目标物的子离子信息。选取2个相对丰度强且稳定的子离子与对应母离子组成定性离子对和定量离子对,采用MRM模式优化选定离子对的去簇电压和碰撞能量,最终确定1.4.2节所述的质谱条件。

### 2.2 样品前处理条件的优化

#### 2.2.1 提取条件的优化

硝基咪唑类药物为酸碱两性化合物,苯二氮卓类药物多为弱碱性化合物,这两类药物在酸性条件下易分解,主要以质子化形式存在,在碱性条件下较稳定,主要以分子状态存在。提取溶剂的极性和pH值对目标物的萃取回收率具有很大的影响。研究表明,动物源食品中硝基咪唑类和苯二氮卓类药物常用的提取溶剂为乙酸乙酯^[14,20]^、乙腈^[15,16,18,26]^、碱化乙酸乙酯^[17,27]^和碱化乙腈^[21,22,28]^等。本实验重点考察了乙酸乙酯、乙腈、1%(v/v)氨水乙酸乙酯和1%(v/v)氨水乙腈对目标物提取效率的影响。结果表明,采用乙酸乙酯和乙腈作为提取剂时,目标物的回收率分别为64.2%~86.7%和68.1%~88.6%;采用1%(v/v)氨水乙酸乙酯和1%(v/v)氨水乙腈作为提取剂时,各目标物的回收率均可达到80%以上,提取效率总体优于乙酸乙酯和乙腈,这说明碱性条件下目标物多以分子状态存在,更容易被有机溶剂提取。同时,实验还发现,采用乙酸乙酯和1%(v/v)氨水乙酸乙酯作为提取剂时,样品提取液中脂溶性杂质较多,溶液较混浊,为后续净化带来困难。因此,综合考虑,本实验选择1%(v/v)氨水乙腈作为提取溶剂。

#### 2.2.2 净化条件的优化

在样品前处理过程,加入氯化钠有利于提取溶剂和水相分层,防止样品中的水分和水溶性极性基质干扰物进入提取液中,从而减少对目标物的干扰和质谱离子源的污染^[15]^;加入无水硫酸钠可以去除提取液中的水分,使浓缩复溶后的样品溶液体积更准确,从而提高目标物的回收率。本实验选用氯化钠作为盐析剂,无水硫酸钠作为除水剂并优化其用量,结果表明,加入1 g氯化钠和3 g无水硫酸钠时,样品提取液具有最好的盐析效果,浓缩后离心管中未发现水分残留,各目标化合物的提取效率较高,回收率为79.6%~102.5%。

为了减少基质效应,提高净化效率和萃取回收率,本研究采用分散固相萃取作为净化手段。吸附剂的选择是分散固相萃取的一个最重要的参数。C18、PSA以及石墨化炭黑(GCB)是常用的吸附剂。其中,C18对脂肪等非极性干扰物具有较强的吸附作用;PSA可以吸附样品中的色素、糖类、有机酸等干扰物质;GCB能有效去除各种基质中的色素和甾醇类等干扰物,但对含有平面结构的化合物具有较强的吸附作用。水产品基质较为复杂,样品经乙腈沉淀蛋白质后可以去除部分干扰物,但仍可能存在少量的蛋白质、脂肪和色素等杂质;苯二氮卓类药物含有苯环类官能团的结构,故本研究只选择PSA和C18两种吸附剂进行优化。在同一加标水平(2.5 μg/kg)的空白样品提取液中,分别加入3个不同组合的PSA和C18(1: 50 mg C18; 2: 50 mg PSA; 3: 25 mg PSA+25 mg C18)吸附剂净化(结果见图2)。

**图 2:**
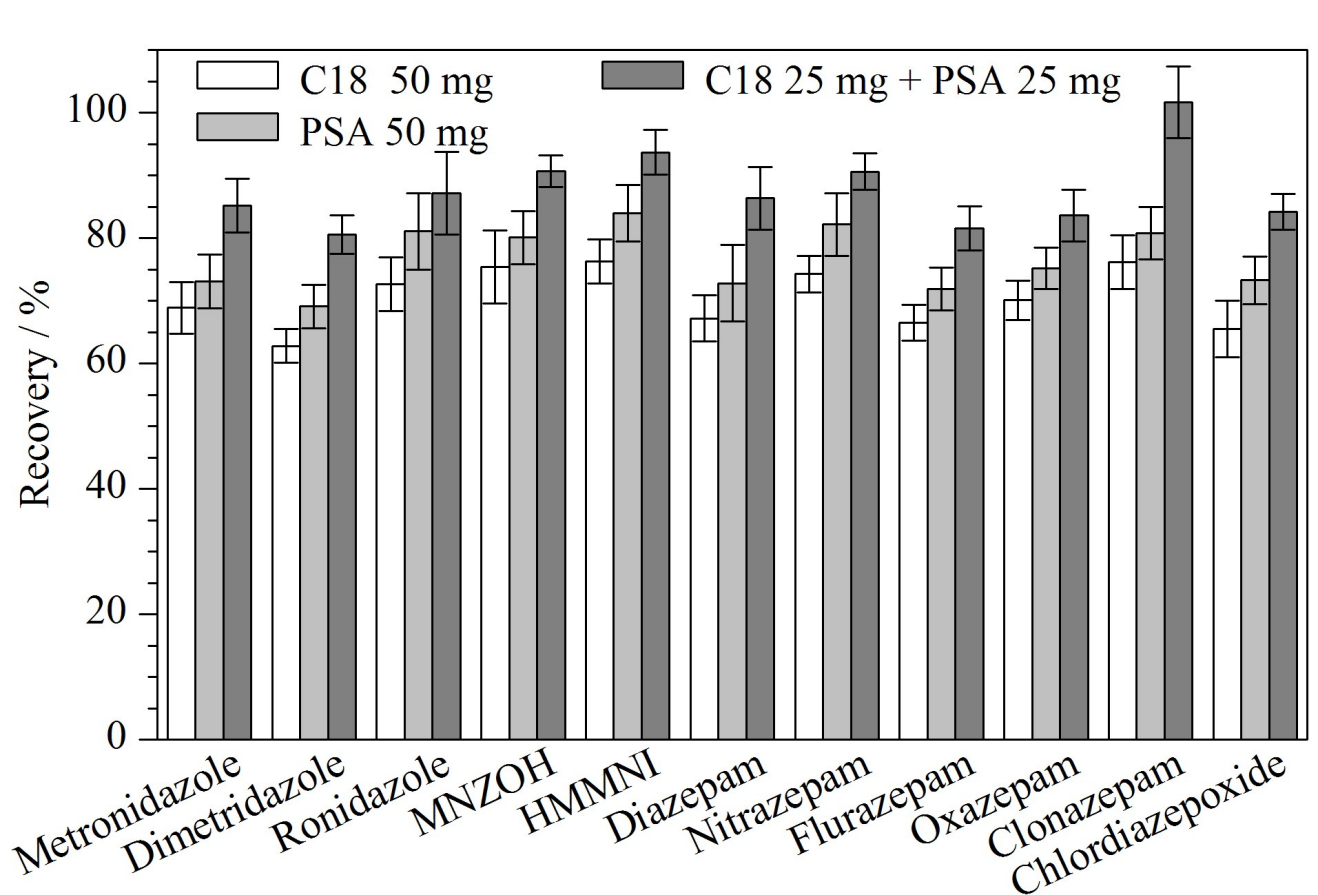
吸附剂类型及用量对5种硝基咪唑类和6种苯二氮卓类 药物回收率的影响(*n*=3)

从图2可以看出,各目标物采用组合3(25 mg PSA+25 mg C18)净化时回收率较高(均大于80%),明显优于组合1和组合2。实验还发现,仅采用C18(组合1)净化时,提取液颜色较深,氮吹浓缩后残渣较多;仅采用PSA(组合2)净化时,氮吹浓缩后脂质残留较多。采用C18(组合1)或PSA(组合2)净化后进样检测,空白样品基线噪声较大,干扰物质较多,质谱的离子源较易受污染且待测物的回收率不理想。实验进一步优化了PSA和C18吸附剂的用量。分别将PSA(25、50、75 mg)和C18(15、30、45 mg)搭配成9个组合进行正交试验,结果发现50 mg PSA+30 mg C18组合的协同净化效果最佳,各目标物的回收率均较高,回收率达到81.5%~103.2%。因此,本实验最终选择50 mg PSA+30 mg C18作为净化吸附剂。

### 2.3 基质效应评价

基质效应(matrix effect, ME)是指在采用LC-MS/MS检测目标化合物时,样品中的基质成分和样品前处理引入的杂质在与待测物离子化过程中相互竞争,影响(增强或抑制)待测物在喷雾接口处的离子化效率,从而导致分析测定结果的准确度和精密度发生改变的现象^[29,30]^。基质效应是LC-MS/MS分析时存在的一个非常显著的现象,一般受分析仪器条件、样品基质类型、前处理方式等因素的影响,会对检测结果的灵敏度、准确度和精密度产生很大影响。因此,在建立LC-MS/MS分析方法时应评估基质效应,并采用适当的方法降低或补偿基质效应的影响,从而确保检测数据的准确、可靠。

基质效应常用的评价方法主要有柱后注射法(定性评价法)^[31]^和提取后添加法(定量评价法)^[32]^。提取后添加法主要有单点提取添加法^[25,28]^和离子抑制率^[33⇓⇓-36]^两种评价方式。本研究采用离子抑制率对基质效应进行评价(见表2),即ME=(*K*_2_/*K*_1_-1)×100%,其中,*K*_1_为溶剂标准曲线的斜率,*K*_2_为空白基质匹配标准曲线的斜率。当ME>0时,称为基质增强效应,表示基质使待测物的响应增加;当ME<0时,称为基质抑制效应,表示基质使待测物的响应降低,当ME=0时表示不存在基质效应。由表2可知,硝基咪唑类和苯二氮卓类药物在草鱼、大黄鱼和对虾基质溶液中存在一定强度的基质增强或减弱效应,ME为-25.8%~12.7%。其中甲硝唑和羟基甲硝唑在3种基质中均存在较强的基质抑制效应,ME为-25.8%~-10.4%。对于基质复杂的水产品而言,经本方法前处理净化后各目标化合物的基质效应均在可接受的范围内。在实际样品测定中,本实验采用空白基质匹配标准曲线定量校准可进一步补偿基质效应的影响。

**表 2:** 5种硝基咪唑类和6种苯二氮卓类药物在不同基质中的基质效应

Compound	Matrix effects/%		
	Grass carp	Large yellow croaker	Prawn
Metronidazole	-25.8	-20.1	-10.4
Dimetridazole	8.9	10.1	-6.2
Ronidazole	5.4	-7.2	-10.5
MNZOH	-15.5	-19.0	-16.9
HMMNI	3.2	-8.5	-4.8
Diazepam	9.1	12.1	6.7
Nitrazepam	0.6	-1.6	-3.4
Flurazepam	12.7	-7.9	-3.6
Clonazepam	1.4	6.3	4.5
Oxazepam	6.6	9.5	-0.5
Chlordiazepoxide	11.5	-12.6	-18.8

### 2.4 方法学验证

#### 2.4.1 线性范围和灵敏度

按1.3节方法配制基质标准溶液,并分别进行测定。以被测组分的峰面积*y*为纵坐标,质量浓度*x*(μg/L)为横坐标,绘制空白基质标准曲线。采用在空白草鱼、对虾和大黄鱼中分别添加低浓度目标物标准溶液的方法,以信噪比(*S/N*)=3和*S/N*=10分别确定各目标物的检出限(LOD)和定量限(LOQ)。5种硝基咪唑类和6种苯二氮卓类药物的线性范围、基质标准曲线、相关系数(*R*^2^)、检出限和定量限见表3。

**表 3:** 5种硝基咪唑类和6种苯二氮卓类药物的线性范围、基质匹配标准曲线、相关系数、检出限和定量限

Compound	Grass carp			Large yellow croaker			Prawn		LOD/ (μg/kg)	LOQ/ (μg/kg)
	Standard curve	R^2^		Standard curve	R^2^		Standard curve	R^2^		
Metronidazole	y=1.64×10^5^x+4.51×10^4^	0.9946		y=1.77×10^5^x+3.52×10^4^	0.9971		y=2.17×10^5^x+4.96×10^4^	0.9956	0.2	0.5
Dimetridazole	y=3.14×10^5^x+2.77×10^4^	0.9973		y=3.18×10^5^x+4.76×10^4^	0.9966		y=2.71×10^5^x+3.75×10^4^	0.9965	0.2	0.5
Ronidazole	y=2.06×10^5^x+1.94×10^4^	0.9964		y=1.82×10^5^x+1.06×10^4^	0.9980		y=1.75×10^5^x+2.55×10^4^	0.9963	0.2	0.5
MNZOH	y=7.97×10^4^x+3.10×10^3^	0.9974		y=7.60×10^4^x+2.14×10^3^	0.9974		y=7.76×10^4^x+6.57×10^3^	0.9974	0.5	1.0
HMMNI	y=1.53×10^5^x+7.34×10^3^	0.9982		y=1.36×10^5^x+5.02×10^3^	0.9963		y=1.41×10^5^x+8.30×10^3^	0.9968	0.5	1.0
Diazepam	y=2.76×10^5^x+2.58×10^4^	0.9979		y=2.84×10^5^x+1.75×10^4^	0.9977		y=2.70×10^5^x+4.36×10^4^	0.9981	0.2	0.5
Nitrazepam	y=3.33×10^5^x+7.22×10^4^	0.9977		y=3.26×10^5^x+5.07×10^3^	0.9981		y=3.20×10^5^x+7.58×10^4^	0.9978	0.2	0.5
Flurazepam	y=1.81×10^6^x+1.44×10^5^	0.9971		y=1.48×10^6^x+2.03×10^5^	0.9982		y=1.55×10^6^x+1.80×10^5^	0.9955	0.2	0.5
Oxazepam	y=3.43×10^5^x+4.60×10^4^	0.9993		y=3.53×10^5^x+3.20×10^4^	0.9992		y=3.21×10^5^x+2.69×10^4^	0.9971	0.2	0.5
Clonazepam	y=2.91×10^5^x+2.48×10^4^	0.9971		y=3.05×10^5^x+1.76×10^4^	0.9990		y=3.00×10^5^x+3.22×10^4^	0.9985	0.3	1.0
Chlordiazepoxide	y=3.86×10^5^x+4.67×10^4^	0.9955		y=3.02×10^5^x+2.69×10^4^	0.9969		y=2.81×10^5^x+3.58×10^4^	0.9976	0.3	1.0

*y*: peak area of analyte; *x*: mass concentration of analyte, μg/L.

由表3可知,5种硝基咪唑类和6种苯二氮卓类药物在0.5~20 μg/L范围内线性关系良好,灵敏度较高。

#### 2.4.2 回收率和精密度

选取阴性草鱼、对虾和大黄鱼作为空白基质样品,设定3个添加水平,每个添加水平做6个平行样,按本方法进行回收率和精密度试验,结果见表4。由表4可知,各基质中5种硝基咪唑类和6种苯二氮卓类药物的回收率为73.2%~110.6%,相对标准偏差为3.5%~14.3%;表明该方法的回收率和重复性好,满足日常检测要求。

**表 4:** 不同基质中5种硝基咪唑类和6种苯二氮卓类药物的回收率和精密度(*n*=6)

Compound	Spiked/(μg/kg)	Grass carp			Large yellow croaker			Prawn	
		Recovery/%	RSD/%		Recovery/%	RSD/%		Recovery/%	RSD/%
Metronidazole	0.5	85.1	3.8		81.2	6.2		81.3	4.9
	1.0	92.3	4.7		90.7	5.0		84.6	5.4
	5.0	88.0	3.5		85.7	4.5		87.0	7.1
Dimetridazole	0.5	82.3	4.0		85.9	4.9		78.8	6.6
	1.0	79.6	3.8		82.0	9.0		85.1	6.4
	5.0	89.8	5.2		90.1	3.8		105.3	6.1
Ronidazole	0.5	79.4	3.6		80.2	3.8		86.6	4.7
	1.0	88.3	4.9		83.5	5.2		80.3	4.1
	5.0	101.5	3.7		91.7	8.0		93.4	10.3
MNZOH	1.0	88.8	7.2		101.2	10.6		83.6	6.9
	2.0	94.6	5.3		105.8	5.4		95.5	6.7
	10.0	102.0	3.9		103.4	4.8		94.1	5.1
HMMNI	1.0	88.2	6.1		93.5	9.2		90.1	7.3
	2.0	93.5	8.0		102.2	9.6		101.4	12.1
	10.0	106.6	3.3		110.6	5.1		103.5	5.4
Diazepam	0.5	83.3	5.6		85.5	5.4		80.0	6.1
	1.0	92.7	3.8		86.1	4.6		84.6	4.0
	5.0	93.8	3.7		101.2	6.2		92.3	4.6
Nitrazepam	0.5	90.7	4.4		86.1	8.1		82.9	7.3
	1.0	87.6	4.5		90.5	5.2		85.4	6.6
	5.0	89.0	5.7		92.2	7.8		84.1	9.4
Flurazepam	0.5	84.6	8.8		79.4	6.4		80.5	12.5
	1.0	85.3	4.2		81.6	3.9		75.6	6.1
	5.0	92.7	6.1		88.1	9.8		82.8	5.0
Oxazepam	0.5	87.2	6.3		81.3	4.6		84.6	10.7
	1.0	95.0	5.9		94.6	8.2		101.3	4.8
	5.0	105.8	2.6		108.2	5.1		110.5	3.7
Clonazepam	1.0	96.1	6.2		103.1	5.1		102.6	4.5
	2.0	85.8	5.1		88.9	6.9		81.7	8.7
	10.0	102.2	4.8		106.2	5.9		97.4	9.6
Chlordiazepoxide	1.0	80.6	9.1		73.2	14.3		78.5	11.1
	2.0	81.3	6.4		84.1	7.1		82.6	5.8
	10.0	86.4	4.7		82.5	5.2		84.3	7.3

### 2.5 实际样品检测

采用已经建立的方法对市售鲫鱼、鳊鱼、鲤鱼、草鱼、鲢鱼、乌鳢、对虾、大黄鱼等30份样品进行分析检测。检测结果显示,所有样品均未检出硝基咪唑类和苯二氮卓类药物。

## 3 结论

本文对仪器检测参数和样品前处理条件进行了优化,采用基质匹配标准曲线外标法定量,建立了一种分散固相萃取-超高效液相色谱-串联质谱法测定水产品中5种硝基咪唑类和6种苯二氮卓类药物残留的分析方法。该方法灵敏度高,回收率好,简单、高效、快速、成本低,实现了对水产品中多种硝基咪唑类和苯二氮卓类药物的同时分析检测,为检测水产品中硝基咪唑类和苯二氮卓类药物残留提供了技术支持。
